# Improving Access and Ability to Age in Place? Effects of Light Rail Transit on Neighborhood Workers and Demographics

**DOI:** 10.1093/geroni/igaf122.1819

**Published:** 2025-12-31

**Authors:** Amber DeJohn, Matthew Suandi, Matthew Palm

**Affiliations:** Florida State University, Tallahassee, Florida, United States; Charles River Associates, Washington, District of Columbia, United States; The University of North Carolina at Chapel Hill, Chapel Hill, North Carolina, United States

## Abstract

Public transit may be a key intervention to address declining mobility in later life. Access to transit is related to health and well-being among older adults; however, older adults have relatively lower transit ridership compared to younger cohorts. Transit projects inherently reshape the built environment, influencing access to community amenities that support aging in place. Disentangling the true consequences of transit construction from pre-existing trends is challenging, since planners design transit systems based on political and economic considerations. While older adults may be a factor in transit planning, it remains unclear whether new transit infrastructure attracts or displaces them. To overcome reverse causality challenges, we identify counterfactual station locations, or alternative alignments, specified by planners in Environmental Impact Statements. These alignments then constitute control areas as part of a difference-in-differences estimation strategy. We include extensions associated with light rails systems opening since 2009. Ultimately, we find that transit investments may primarily serve working older adults, but retirees may be deterred from these neighborhoods. Specifically, the construction of light rail causes the proportion of older adults to decline in the immediate, walkable area of the new station. This effect is particularly significant in larger metropolitan areas included in our analysis, like Los Angeles. When considering the number of workers over 55 who live near newly built stations, we find a growing population as well as increasing numbers of workers with high salaries. In sum, not all older adults reap benefits from new transit investments.

